# Stem Cell Therapy and Rejuvenation, and Their Impact on Society

**DOI:** 10.3390/bioengineering10060694

**Published:** 2023-06-07

**Authors:** Gaskon Ibarretxe

**Affiliations:** Department of Cell Biology and Histology, Faculty of Medicine and Nursing, University of the Basque Country (UPV/EHU), 48940 Leioa, Spain; gaskon.ibarretxe@ehu.eus; Tel.: +34-946-013-218

## 1. Introduction

In his worldwide best-seller *Homo Deus* [1], historian and philosopher Yuval Noah Harari discusses the future challenges facing humankind. In particular, he predicts that humanity will pursue three goals in the coming decades: immortality, bliss, and divinity. Biomedical engineering is pivotal to at least two of these alleged human aspirations—immortality and divinity—and it may arguably pertain to the pursuit of bliss, as well. The possibility of replacing or upgrading cellular constituents of the human body has led to an unprecedented scenario; for the first time in history, new cells and body tissues can be generated ex vivo to replace damaged or decayed body parts via cell transplant, similarly to the replacement of pieces of a LEGO^®®^ figure. The use of stem cells for tissue/organ reconstruction and for the treatment of lesions and diseases has seen overwhelming success over the past few decades, and multiple well-validated therapies are now available for dealing with hitherto incurable conditions. These stem cell therapies may rely on both genetically modified and primary cells. Many, but not all, stem cell therapies fall into the category of biomedical engineering because they may include genetic manipulation, as well as sophisticated scaffold materials, innovative cell culture and delivery systems, and cell imaging and monitoring systems. With the abundant public and private funding contributed to achieving this goal, the field of biomedical engineering is bound to grow substantially in the future. Furthermore, in the next few decades, researchers may even refine stem cell and tissue manipulation technology to such an extent as to prevent or delay cellular decay associated with aging, which brings us to the concept of cell rejuvenation. Although we are arguably still quite far from the point of generating eternally young, a-mortal humans, many renowned scientists are fully dedicated to studying the mechanisms of cellular aging and rejuvenation, many of whom are hired by private biotechnology companies with incredibly large amounts of funding available. This editorial seeks to discuss the main features, advantages, and limitations of stem cell therapies in combination with cellular rejuvenation strategies, and their impact on society. Paradoxically, despite its role in addressing important challenges that could influence the future of humankind, the social impact of stem cell therapy research, measured in terms of improved healthcare outcomes, will only become evident many years, or even decades, after it has been tested in experimental models at the laboratory level.

## 2. Stem Cell Therapies for Tissue Healing and Regeneration

Stem cells possess two distinct features: (i) self-renewal, or the ability to sustain successive rounds of cell division, and (ii) potency, or the ability to give rise to other specialized cell types. According to this principle, stem cells can be classified as totipotent, pluripotent, multipotent, or unipotent [2]. Most mature cells in the human body are highly specialized (differentiated) and have lost their ability to undergo cell division; hence, they are also referred to as “post-mitotic cells”. When differentiated post-mitotic cells die as a result of natural aging or lesions/disease, they must be regenerated through the activation of local stem cells, which are often tissue-specific and restricted to particular niches in the adult body [2,3]. Adult tissues vary greatly in their self-renewal rates, with some tissues harboring an abundant, highly proliferative population of stem cells (for example, the gastrointestinal and skin epithelia and bone marrow) whereas others contain relatively quiescent stem cell populations that can be activated in response to injury, such as muscles and the central nervous system [4]. Natural tissue renewal depends on these adult tissue-specific stem cells, which can be harvested for ex vivo expansion, and the development of Tissue Engineering Therapies [5]. Alternatives to adult-tissue stem cells are embryonic stem cells (ESCs) and induced pluripotent stem cells (iPSCs), but their use in regenerative therapies is largely limited by ethical and safety constraints, respectively [6,7].

Since the discovery of bone marrow stem cells as the healing component of bone marrow transplants against hematological disorders in the 1960s, the stem cell research field has expanded considerably, and it is expected to continue to do so in the future. Many excellent review articles have been published in this journal over the last few years, showing progress in the use of stem cells for the treatment of a variety of diseases and conditions, such as *Diabetes Mellitus* [8], ischemic stroke [9], infectious diseases [10], and wounds [11]. The benefits of stem cell therapy include both cell replacement and paracrine mechanisms. A prevalent line of research at the moment is the use of stem cell-secreted extracellular vesicles, which avoid most of the major risks associated with long-term cell transplantation [12]. Stem cells can also be genetically modified for a particular application, such as curing a congenital disease [13]. This brings up the issue of stem cell modification and enhancement, with potential applications that could extend beyond their use in disease therapy.

## 3. Stem Cell Rejuvenation

One obvious problem with stem cell therapies is the onset of cellular senescence, in which cells lose their ability to divide and self-renew. This phenomenon is closely related to aging, and may have different causes, such as excessive accumulated population doubling (replicative senescence) and genotoxic, oxidative, and/or metabolic stress [14,15]. In addition, senescent cells show an altered secretome, which can have deleterious effects on tissues [16]. Stem cell senescence is a major limiting factor in tissue engineering, and several rejuvenation strategies have been devised to prevent or delay it. Some of the most effective rely on the genetic overexpression of “rejuvenating factors”, such as the widely known Yamanaka Factors OCT4, SOX2, KLF4, and CMYC (OSKM); Telomerase Reverse Transcriptase (TERT); or other transcription factors such as NANOG, YAP, and FOXD1 [15,17]. This approach has even been applied in vivo, where rejuvenation has been shown in different organs and at many levels following the forced overexpression of OSKM and other pluripotency-related transcription factors in transgenic mice [18,19,20,21].

Similarly to the development of artificial intelligence, the possibility of rejuvenating aging tissues and artificially extending the human lifespan could open up a Pandora’s box for humanity, with a myriad of both promising and questionable potential uses and implications. However, there is a limitation. At their current stage of development, these rejuvenation strategies depend on permanent genetic modification, which carries a high risk of side effects. For instance, Yamanaka factors are well-known for their reprogramming function, but they are also known to be highly oncogenic [22]. Thus, one major issue pertains to the cyclic induction of these rejuvenating factors; researchers must restrict their expression to the minimum required amount for the shortest possible duration and shut them off immediately afterwards using drug-inducible promoters [18,19,20].

Leaving aside the risks of genetic rejuvenation, other less radical approaches have been experimentally developed to enhance stem cell renewal and potency while delaying senescence. Those include the stimulation of autophagy [23,24] and hypoxia [25,26], and the pharmacological regulation of different signaling pathways such as mTOR [15,23], PI3K/AKT [27,28], or Wnt/β-catenin [29,30]. Notably, these treatments induce changes at the epigenetic level, indirectly resulting in the higher expression of OSKM and other reprogramming factors by stem cells, but with fewer side effects than traditional reprogramming methods. The limitation is that these pharmacological and/or metabolic interventions may induce a more subtle and much less consistent rejuvenation phenotype than outright genetic OSKM overexpression. Regardless of the rejuvenation methodology chosen, a final constraint that has received relatively little attention so far is the accumulation of somatic mutations in stem cells. If stem cells are artificially induced to bypass senescence mechanisms, one likely long-term consequence is the induction of tumorigenesis [31]. After all, senescence is a natural mechanism intended to prevent the propagation of malignant or irreversibly damaged cells. It is unclear how somatic gene mutations associated with normal aging might interact in the context of the cellular overexpression of reprogramming factors.

Nevertheless, research continues, and the understanding of the aging process and the development of new strategies for organism rejuvenation are the objectives of powerful and generously funded private companies such as Rejuvenate Bio (https://rejuvenatebio.com/), Calico Labs (https://www.calicolabs.com/), and Altos Labs (https://altoslabs.com/). Given the importance of the issue at hand, it is possible that these and other related research companies will continue to fare well in the future. In any case, it is likely that if an “organism rejuvenation therapy” becomes available, it will be restricted to the very wealthy, given the high costs associated with these technologies.

## 4. Research on Stem Cell Therapies: The Long-Term Social Impact

Those familiar with stem cell-based therapies may understand the long and difficult road that precedes a promising discovery at the laboratory level, at which point researchers will seek to translate it to clinical practice in the form of an Advanced Therapeutic Medicinal Product (ATMP). In their review article published in *Bioengineering* in 2022, Muthu et al. [32] present many of the regulatory challenges that apply to such cases, including the funding of extremely expensive clinical trials under Good Manufacturing Practice (GMP) laboratory standards. The reason for this regulatory complexity is that we are dealing with the most complicated and unpredictable medicaments of all: live cells and tissues. Paradoxically, this difficulty regarding translation into clinical practice could constitute an obstacle to the social recognition of this research, since few of the many stem cell therapies that are developed at the experimental level reach the clinic and are translated into effective improvements in the clinical management of patients. Additionally, the experimental therapies that do succeed take a long time to reach patients.

Impact assessment in health sciences is an important process for assessing the effectiveness and usefulness of biomedical research in improving healthcare outcomes. Accordingly, research on issues such as organ regeneration and rejuvenation could be regarded as having major potential health impacts. However, stem cell research produces results regarding social impact over a relatively long time scale of many years, or even decades. Thus, it is important to also consider the context and purpose of the research being assessed [33,34]. We must recognize and promote the interconnectedness of preclinical and clinical research as a major way to generate a meaningful social impact, as advances in preclinical research often pave the way for clinical trials and the development of new therapies. Unquestionably, research on stem cell therapies combined with cell rejuvenation could have a substantial long-term impact on society.

## 5. Conclusions

There is no doubt that stem cell therapies combined with biomedical engineering and cell rejuvenation could have a considerable and permanent impact on humanity. Arguably, the process of developing rejuvenation therapies is in its infancy, and it could take a long time for them to become a reality. However, the same was once true of other diseases and lesions that can be now cured using stem cell transplants. Downplaying the social impact of stem cell therapies may affect public resource allocation to this field, but such research will continue nonetheless with the support of private funding.
